# Diagnosis of Bovine Genital Campylobacteriosis in South America

**DOI:** 10.3389/fvets.2018.00321

**Published:** 2018-12-14

**Authors:** Caroline da Silva Silveira, Martin Fraga, Federico Giannitti, Melissa Macías-Rioseco, Franklin Riet-Correa

**Affiliations:** ^1^Instituto Nacional de Investigación Agropecuaria (INIA), Plataforma de Salud Animal, Estación Experimental INIA La Estanzuela, Colonia, Uruguay; ^2^Veterinary Population Medicine Department, College of Veterinary Medicine, University of Minnesota, Saint Paul, MN, United States

**Keywords:** Venereal Bovine Campylobacteriosis, infertility, abortions, *Campylobacter fetus* subsp. *venerealis*, diagnostic methods, South America

## Abstract

Bovine genital campylobacteriosis (BGC) is a venereal infectious disease that affects reproduction. It is caused by the Gram-negative bacillus *Campylobacter fetus* subspecies *venerealis* (*Cfv*), which may include the biotype intermedius. The bull is a lifelong asymptomatic carrier and transmitter of the disease. In females *Cfv* may cause infertility and sporadic abortion. The objective of this study is to review and discuss methods for the diagnosis of BGC, its prevalence and economic impact in South America. BGC is a worldwide distributed disease and can cause a pregnancy rate decrease of 15–25%. The farm prevalence of BGC in different regions of South American countries shows a variation between 2.3 and 100%. Discrepancies may depend on the differences on sanitary, management, and reproductive practices between farms and regions, but also on the interpretation of different diagnostic tests. Currently known laboratory tests include bacterial culture, direct immunofluorescence, immunoenzymatic assays, vaginal mucus agglutination test, PCR-based methods, histology and immunohistochemistry, which are applied and interpreted in diagnostic laboratories at different scales. Epidemiologic data of BGC in South America should be interpreted with caution. High prevalence has been reported in some studies, although the low specificity of the diagnostic tests used could lead to an overestimation of the results.

## Introduction

Reproductive diseases of cattle resulting in infertility and abortion cause significant economic losses (1). On average, a specific cause can only be identified in ~45% of the aborted bovine fetuses examined in veterinary diagnostic laboratories in South America. The proportion of abortion cases attributed to *Campylobacter* spp. ranges from 0 to 13% of all fetuses with a confirmed etiology (2–6). Similar values are found in other countries, such as the USA, where a confirmed diagnosis is reached in 35.25% of the cases, and *Campylobacter* spp. accounts for 1.8–10.6% of the cases with an etiologic diagnosis (7). Venereal diseases, such as bovine genital campylobacterosis (BGC) caused by *Campylobacter fetus* subspecies *venerealis* (*Cfv*) (8), are important causes of these reproductive losses. *Campylobacter fetus* is a curved, motile, non-spore forming, Gram-negative bacillus with one or two polar flagella. The species comprises the subspecies *Cfv*, which may include the biotype intermedius (9, 10), and the subspecies *Campylobacter fetus fetus* (*Cff* ) and *C. fetus testudinum* (*Cft*) (11). *Campylobacter fetus fetus* is a common resident of the mammalian intestine and can cause abortion in cattle and sheep (12), as well as enteric or systemic disease in humans (13). Transmission of *Cff* occurs mainly via the fecal-oral route; this is followed by a transient bacteremia during which, in pregnant ruminants, the agent can translocate to the placenta resulting in placentitis and abortion (14). *Cft* is not clinically important for cattle, it infects in reptiles and has also been isolated from humans (11).

Bovine genital campylobacteriosis was first recognized as a cause of infertility in cattle in the 1940s and was known to have worldwide distribution in the 1960s (15). In South America it was first diagnosed in 1955 in Brazil, through the isolation of the bacterium from an aborted bovine fetus (16). Bovine genital campylobacteriosis is an important cause of reproductive failure, infertility and irregular estrus in both heifers and cows, although heifers are more susceptible to infection due to the low levels of immunity (17–19).

Herds with *Cfv* infection often have reduced breeding efficiency, including pregnancy rates lower than expected, embryonic losses, sporadic abortions, increased number of services per conception, extended calving season, and a longer interval between calving. Abortions are more commonly detected between the 4th and 6th months of gestation (15, 20–23). The average pregnancy rate in infected herds varies depending on the relative proportion of carriers, susceptible and immune breeding-age females, and infected and non-infected bulls (21, 24).

Bulls are the most important reservoirs and disseminate the disease through coitus (9). *Campylobacter fetus venerealis* lodges in the preputial crypts; the hosts do not develop any clinical signs or lesions due to this infection, acting as asymptomatic carriers. Infection does not affect libido or sperm quality (9, 20). For this reason, bulls should be the target category for diagnostic approaches, epidemiological studies, as well as control and prevention strategies. After venereal transmission in females, infertility associated with *Cfv* infection occurs due to endometritis that causes changes in the uterine environment that interfere with embryo nesting. In addition, the inhospitable environment can also affect embryos that are already implanted and fetuses (25).

Dairy and beef production play an important role in the economy of South American countries. Brazil, Argentina, and Uruguay are the three countries with highest cattle stock in the region, reaching 172, 57, and 13 million heads in 2016, respectively (26–28). The awareness of the socioeconomic importance of this sector, and the economic impact of venereal diseases of cattle (BGC and trichomonosis), led in 2006 to the implementation of the Provincial Control and Eradication Program (PCEP) of bovine venereal diseases by the Ministry of Production of the province of La Pampa, Argentina, together with the College of Veterinarians, the National Institute of Agricultural Technology (INTA) and the National Service of Sanitation and Agri-food Health and Quality (SENASA). Engagement in the program is mandatory for all herds in La Pampa, testing should be performed prior to cattle movements. All non-virgin bulls are tested twice a year, and positive bulls are culled within 120 days of diagnosis (testing and culling strategy). The PCEP proved effective to reduce the prevalence of these diseases from 2007 to 2014 in the province (29), providing a framework to set up sampling procedures and testing methodologies to identify infected bulls more efficiently. However, there are intrinsic difficulties with the laboratory diagnosis of BGC in the region that have yet to be overcome to establish more effective control strategies (30).

The diagnosis of BGC presents significant challenges. The objective of this study is to review and discuss methods for the diagnosis of BGC, and its prevalence and economic impact in South America.

## Diagnosis OF BGC

### Sample Collection

For the diagnosis of BGC, various methods that demonstrate either the presence of *Cfv* (i.e., bacterial culture) or some of its components (i.e., DNA, proteins), or host-developed immune response against *Cfv* (immunologic assays) have been developed (8) (Table 1). The quality of the samples submitted for diagnostic testing is extremely important because it directly influences the accuracy of the results obtained (31).

**Table 1:** Available techniques for the diagnosis of BGC.

**Diagnostic technique**	**Samples**	**Time to diagnosis**	**Detectable infection**	**Diagnosis of BGC**
Bacterial culture and phenotypic identification	Preputial smegma; vaginal mucus; fetal tissues and fluids	7–10 days	*Cfv; Cff*	Variable
DIF	Preputial smegma; vaginal mucus; fetal tissues and fluids	1 day	*C. fetus*	No
Antigen capture ELISA	Preputial smegma; vaginal mucus	5–6 days	*C. fetus*	No
PCR-based methods	Preputial smegma; vaginal mucus; fetal tissues and fluids	4–8 h	*Cfv; Cff*	Yes
IHC	Fetal tissues	3 days	*C. fetus*	No

*BGC, Bovine genital campylobacteriosis; DIF, Direct Immunofluorescence; Cfv, Campylobacter fetus venerealis; Cff, Campylobacter fetus fetus; IHC, immunohistochemistry*.

Because bulls are lifelong asymptomatic carriers, and disseminators of the disease, they are the animals of choice for the diagnosis of BGC in endemic herds (21). *Cfv* establishes persistent colonization of the preputial crypts, which may be linked to the modification of the molecular composition of bacterial surface antigens that allow it to escape the local immune response (32). Given the ecologic niche of *Cfv*, diagnostic samples for detecting or isolating the agent should preferably be taken from the prepuce of bulls.

For this purpose, smegma may be obtained from the preputial and penile mucosa by three different methods: (A) scraping: performed by scarifying the foreskin and penile mucosa using a disposable plastic or sterilizable reusable metal scraper (33) that is then rinsed in phosphate buffered saline (PBS); (B) aspiration: performed through a disposable plastic sheath coupled to the artificial insemination pipette to suction the smegma; and (C) washing: performed by introducing about 20–30 mL of PBS into the foreskin, massaging it with the closed ostium before collecting the material through a siphon system (8, 34). Preputial scraping, compared to the other two methods, is the technique of choice because more *C. fetus*-positive samples are identified when samples are obtained by this method (33). McMillen et al. (35) confirmed that the scraping method is more effective in recovering *Cfv* than the other two collection methods. In addition, the authors emphasize that this technique is easier and safer to perform.

Bulls should be kept in sexual rest for 15 days before sampling, and three sample collections, with the same resting interval, should be performed to increase the diagnostic sensitivity (34). Downsides of this practice include that A-the bulls need to be out of service for ~45 days, and B-multiple visits to the farms are required to complete sampling, which implies greater logistic efforts by both veterinarians and farmers.

In contrast, cows and heifers infected with *Cfv* are temporally colonized by this pathogen, thus females are not usually sampled and tested for herd screening purposes. However, when gestational losses occur, the cervicovaginal mucus and placenta from aborted dams, and fetal organs and fluids represent adequate diagnostic samples (3, 21). The same plastic sheaths coupled to the artificial insemination pipette used in bulls, can be successfully used in dams to suction cervicovaginal mucus. This represents an easy and practical sampling method. Eventually, a sterile speculum can be used to collect a cervicovaginal washing (8, 34, 36).

Current BGC diagnostic methods include: A-tests that either demonstrate the presence of *Cfv* (i.e., bacterial culture) or its components (i.e., direct immunofluorescence or immunohistochemistry for the detection of surface proteins, PCR-based methods for detection of DNA), or B-tests that aim at detecting host-developed immune response against *Cfv* (i.e., immunoenzymatic assays, vaginal mucus agglutination test) (3, 8, 22, 31, 37–41).

### Bacterial Culture Approaches

Genital secretions (preputial smegma and cervicovaginal mucus), placenta and fetal fluids (i.e., abomasal content) and/or tissues (i.e., liver and lung) represent adequate samples for isolation and further identification of *Cfv*. In cases where BGC is suspected, samples should be collected aseptically and transported to the diagnostic laboratory (8, 34). To increase the chances to isolate this fastidious microorganism, cultures should be carried out within 4 h of sample collection (42). However, the use of proper transport media extends this period to about 24 h (42). Transport and enrichment media (TEM) are available to optimize culture, such as Weybridge, Cary-Blair, Clark, Thomann, Lander, and 0.85% sterile saline solution (8, 43–45). Usually, Weybridge TEM is the medium of choice, as it is efficient in maintaining viable *Cfv* and reducing contamination (44). Thomann TEM has proved effective for both culture and PCR approaches (45).

Physiological characteristics of *Cfv* makes laboratory culture difficult as this microaerophilic and fastidious microorganism requires special growth conditions. Procedures for isolating *Cfv* involve the use of enriched culture media with antibiotics to minimize the growth of contaminants, and incubation in microaerobic conditions (5–10% O_2_, 5–10% CO_2_, preferably 5–9% H_2_, and the rest of N_2_), at 37°C for a minimum of 48 h (8, 34).

Enriched culture media such as Skirrow agar, bright green agar, and blood agar are recommended for *Cfv* isolation. When managing microbiologically complex clinical samples, such as preputial smegma, Skirrow agar is the best choice (44). This medium contains inhibitors that minimize the growth of undesirable contaminating microorganisms, thus facilitating the development of *Cfv* and the consequent observation of compatible bacterial colonies (44). Passive filtration of the sample onto the media can increase the recovery of *Cfv* and reduce fungal contamination (42). Additionally, Skirrow could also be used for the isolation of *Brucella* spp., pathogens that share niche and are present in South America (46).

The morphologic characterization of the bacterial colonies is not sufficient for the identification of *Campylobacter* to the species and subspecies levels. For this reason, it should be considered that *Cff*, which is also clinically important but not related to BGC, can grow in the same selective media and show similar colony characteristics as *Cfv*. These subspecies should be distinguished by their phenotypic characteristics based on biochemical tests (8, 21) or molecular approaches (see section on PCR-based methods below). While *Cff* produces H_2_S and is able to grow in the presence of 1% glycine and 0.1% sodium selenite, *Cfv* does not (8, 12). However, there is concern about the reliability of these biochemical characteristics for definite subspecies identification, as some *Cfv* strains had acquired the glycine tolerance characteristic. Chang and Ogg (47) described that this process occurred by transduction and mutation events. In addition, a group of *Cfv* denominated biovar intermedius also, as *Cff*, produces H_2_S, which may lead to reaching erroneous conclusions regarding the identification of *Cfv* (10, 12).

### Direct Immunofluorescence (DIF)

Direct immunofluorescence is widely used for the diagnosis of BGC on samples of preputial smegma, cervicovaginal mucus, uterine tissue, placenta, and abomasal fluid, lung and liver from aborted fetuses (3, 22, 31, 36, 41, 48, 49), and is listed by the OIE as a prescribed diagnostic method for international bull trade (8). This test proves to be very effective even in contaminated field samples. The detection limit is 10^4^ and 10^2^ *Cfv* CFU/mL in non-centrifuged and centrifuged preputial washings, respectively (31).

For DIF, samples should be stored in PBS with 1% formalin after collection. Genital fluid samples should be centrifuged to remove debris and contaminating particles. The fluid is placed on glass slides and subsequently fluorescein isothiocyanate conjugated (FITC) antiserum is added in the appropriate dilution. The slides are examined under ultraviolet light in a fluorescence microscope. Samples showing fluorescent bacteria with the typical *C. fetus* morphology are considered positive (8, 31).

A study performed in Argentina evaluating preputial smegma and cervicovaginal mucus through DIF determined a sensitivity and specificity of 69.4 and 94.4%, respectively (36). A similar study in Brazil estimated a sensitivity of 92.59% and a specificity of 88.89% (31). The adequate interpretation of the slides may be challenging, especially when there are contaminating particles and cellular debris with low concentration of *Cfv* in the fields of microscopic observation (31, 41). The performance of this technique is directly influenced by the quality of the sample and the microscope used, and also by the observer's experience (50), which can lead to a reduction in test sensitivity. Therefore, adequate training is key in diagnostic settings. Contrary to this statement some authors consider that the effect of experienced observers on test performance is minimal (31).

Routine DIF protocols generally employ polyclonal rabbit raised FITC antibodies that do not discriminate between the two *C. fetus* subspecies (8). There is a risk for false positive *Cfv* results when the sample contains *Cff* (8, 31), therefore caution should be taken when interpreting DIF results alone. Chicken raised antibodies (IgY) against *C. fetus* can also be employed in DIF protocols. Although IgY based protocols had similar sensitivity when compared with rabbit IgG they show lower unspecific background fluorescence and are cheaper to produce (48).

However, DIF is rapid and advantageous compared to more time-consuming assays for *C. fetus* testing. Using samples of vaginal mucus, Marcellino et al. (41) compared the diagnostic efficiency of the DIF technique with the bacteriological culture, obtaining a moderate agreement (kappa coefficient = 0.52) between these techniques. However, usually vaginal mucus samples do not harbor as many microbial contaminants as preputial samples. Considering that *Cff* and *Cfv* are both subspecies of clinical importance and may both cause reproductive losses, DIF is an easily applied technique suitable for the screening of *C. fetus* in endemic BGC herds and abortion episodes. However, for the definitive diagnosis of BGC, more specific and sensitive tests are needed to provide evidence of *Cfv* infection.

Currently, DIF is widely used in most diagnostic laboratories in South America as the only diagnostic test for BGC, possibly because cultivation is a difficult technique to use for routine testing, and PCR protocols are not yet standardized and validated under South American conditions. The lack of experience, equipment and funding required to conduct more sensitive and/or specific tests is still a limiting factor in most veterinary diagnostic laboratories in the region.

### ELISA and Other Immunologic Assays

An enzyme-linked immunosorbent assay (ELISA) was developed to detect secretory IgA antibodies specific for *Cfv* antigen in the vaginal mucus and used in the diagnosis of *Cfv* induced abortion (51). In infected cows, especially those that aborted recently, there is a strong immune response of local antibodies in the vaginal and uterine mucosa (51). This test has been used to screen for BGC in cattle herds with infertility and abortions; the specificity was found to be 98.5%, although sensitivity could not be estimated (43). It has been postulated that vaccination against campylobacteriosis does not interfere with the IgA ELISA test, as only IgG, but not IgA, is secreted in the vaginal mucus of vaccinated cows (43). As the immune response in the preputial mucosa of bulls carrying the bacterium is fleeting (32), tests aiming at detecting antibodies in preputial smegma should be avoided.

ELISA tests have also been developed for the detection of *C. fetus* antigens in enriched bacterial cultures. In a study, field samples including preputial washings, placenta from aborted cows, and abomasal fluid from aborted fetuses, were incubated for 4 days in Clark's TEM and then tested with a monoclonal antibody-based antigen capture ELISA for the detection of *C. fetus* as a screening test. The sensitivity and specificity were of 100 and 99.5%, respectively, compared to conventional culture (52).

An indirect ELISA for the detection of serum antibodies against *C. fetus* was developed with rSapA-N, a recombinant protein codified by the *C. fetus*-specific virulence gene *sapA*. The specificity and sensitivity of this method were 94.3 and 88.6%, respectively (53). This test does not discriminate between antibodies against the two *C. fetus* subspecies.

ELISAs can be used as a first screening of herds with *C. fetus* infection, allowing for rapid large-scale sample processing (43, 52–54). However, there are no reports of the application of these techniques for BGC diagnosis in South American countries, and commercial kits are not readily available in this region.

The vaginal mucus agglutination test (VMAT), was used in the past decades to detect antibodies in vaginal mucus washes. The sensitivity was ~50% (15, 55). This assay resulted in many false negative results not only because of the intrinsic low sensitivity, but also because antibodies are detected in vaginal mucus after 2 and before 7 months of infection (56). Therefore, the use of VMAT was discouraged. This test has not been widely used with diagnostic purposes in South America.

### PCR-Based Methods

Culture-independent methods such as polymerase chain reaction (PCR) (10), real time PCR (35, 39, 57) and multiplex PCR (37–39, 58) are also used for the diagnosis of BGC. These approaches have improved reported sensitivity and specificity compared to other techniques such as microbiological culture (57). These techniques can detect specific *Campylobacter* DNA sequences by means of the design of specific primers (37, 39, 59).

The differentiation of *Cfv* from other species and subspecies can be carried out through the molecular genotype classification by means of multiplex PCR techniques. In these approaches, specific primers designed to detect *Campylobacter* spp. or the different subspecies are used (37, 38). Another sensitive variation is real time PCR that is theoretically capable of detecting and quantifying DNA from a single *Cfv* cell in field samples (35, 57, 59). PCR-based methods have advantages over microbiological cultures, such as simplicity in interpretation of results. A multiplex PCR protocol targeting sequences present in *Cfv* but not in *Cff*, was developed in Uruguay (39). Interestingly, genomic DNA is directly extracted from the abomasal fluid of aborted bovine fetuses and the downstream process involves only one step (39). Applying this protocol, *Cfv* biovar intermedius could be detected when cultures and biochemical tests were not consistent (39, 60, 61). However, a recent study demonstrates that the *virB11* gene, used as a target gene for differentiation between subspecies in the above-mentioned PCR protocol (39), is not exclusively present in *Cfv* strains and is not always absent in *Cff* strains (62).

The complete genome of an Argentinian *Cfv* biovar intermedius strain was sequenced (61). The genomic information of such atypical strains would optimize the development of tools for more specific molecular diagnosis. Recently, the study of two genomes, reported incongruities between the *Cff* and *Cfv* lineages and the biochemical characteristics used for their differentiation, questioning the clinical relevance of subtyping mammalian strains (63, 64).

Van der Graaf-van Bloois et al. (60) tested five PCRs that are routinely used by diagnostic laboratories, and surprisingly none of them were able to correctly identify strains of *C. fetus* at the subspecies level. All the tests were compared with the methods of amplified fragment length polymorphism (AFLP) (65) and multilocus sequence typing (MLST) (10), which so far are the only techniques that have proven to reliably differentiate the two subspecies. Unfortunately, these assays are cumbersome, costly and impractical for routine use.

The differentiation of *C. fetus* subspecies is essential for the implementation of efficient *Cfv* control and eradication programs, and for investigating the public health burden of *C. fetus* subspecies, however its genomic evolution in mammals remains poorly understood. A recent study provides the phylogenetic and evolutionary structure of *C. fetus* which could guide the development of methods for differentiation and epidemiological surveillance of bovine and human strains (66).

In South America, even though molecular tests have been developed and used in recent years, their applicability to routine laboratory diagnosis is currently subject to discussion. This is due to the discrepancy between results obtained by different protocols, and because most protocols cannot be performed with confidence directly on DNA extracted from field samples (i.e., preputial smegma). These factors have led so far to the non-acceptability of the technique by field veterinarians and diagnostic laboratories. Even though it is likely that these technologies will be increasingly adapted by veterinary laboratories in years to come, particularly considering that they are accepted by the OIE standards (8). Phenotypic tests are still the only ones that are reliable and available in South America for the identification of subspecies. However, they are poorly reproducible and do not correspond to the genomic characteristics of some strains (63, 64). Due to these aspects, further research is needed to validate molecular techniques with locally isolated strains and to assess their applicability in the region.

### Histochemical and Immunohistochemical Methods

Histology and immunohistochemistry are useful and effective diagnostic methods in natural cases of bovine abortions by *C. fetus* (22), particularly in fetuses and placentas from which *C. fetus* cannot be successfully isolated because of poor preservation of the samples (i.e., aborted fetuses that are autolytic, frozen or deteriorated upon arrival to the laboratory) (23).

The main histologic lesions in aborted fetuses are suppurative pneumonia, myocarditis, fibrinous serositis, interstitial nephritis, hepatitis, gastroenteritis, meningitis, and necrotizing placentitis; however, these lesions are not pathognomonic of *C. fetus* infection (22, 23, 40). Immunohistochemistry for the detection of *C. fetus* antigens in formalin-fixed paraffin-embedded tissues, in the absence of bacterial isolation and molecular evidence of infection, is an alternative and practical method to establish the diagnosis in aborted fetuses (22, 23, 36).

Pathological examination coupled with DIF and/or bacterial culture has been widely used in Brazil, Argentina, and Uruguay for the diagnosis of *Campylobacter*-induced abortion (3–6, 22, 23, 67). However, this does not seem to be a broadly used diagnostic approach in other countries in the region.

## Economic Impact and Prevalence

Bovine genital campylobacteriosis is a disease of worldwide distribution (24). The prevalence is high in developing countries where extensive cattle breeding is widely practiced (21), and the infection status in a herd can be easily overlooked. Bovine genital campylobacteriosis may lead to significant economic losses in countries that maintain reproductive management based on natural mating with bulls, such as Uruguay, Brazil, Argentina and Colombia (68–70).

Studies on beef herds show that the disease can lead to a 15 to 25% reduction in pregnancy rates (2). In dairy herds, *C. fetus* infection negatively influenced milk production (71), however, these losses were not quantified. Considering that the cost of an abortion in dairy cattle in the Unites States has been estimated in approximately $555 per animal (72), a hypothetical reduction of 20% for gestational losses in a herd of 1,000 pregnant cows would represent an economic loss of $111,000.

There is no literature available on the quantification of economic losses caused by BGC in South America. In the last decades high prevalence of BGC has been reported in dairy and beef herds in Brazil (20, 73–79), Uruguay (49), Argentina (29, 36, 80), and Colombia (81). A summary of studies reporting prevalence of BGC in different regions of South American countries between 1984 and 2018 is presented in Table 2. It should be mentioned that most of the studies were conducted with few herds, so it is likely that they do not reflect the true prevalence accurately, as highlighted by Molina et al. (29).

**Table 2:** Studies reporting prevalence of BGC in different regions of South American countries between 1984 and 2018.

**Country**	**Region**	**Diagnostic technique**	**Origin of sample**	**Prevalence**				**References**
				**N^**°**^ farms** **(+/total)**	**Farm** **(%)**	**N^**°**^ animal** **(+/total)**	**Animal** **(%)**
Brazil	MS	DIF^a^	Bull	17/19	-	171/327	51.7	Pellegrin et al. (20)
	MG	DIF^a^	Cow	9/9	100	40/157	25.5	Stynen et al. (73)
	BA, GO, MA, MT, MS, MG, PA, PR, RS, RO, SP e TO	DIF^a^	Bull	224/1191	19.7	61/120	50.8	Miranda (74)
	RJ	DIF^a^ and I^b^	Bull	–/9	-	14 and 4/39	35.9/10.3	Rocha et al. (75)
	RS	PCR^c^	Bull/Cow/ Fetus	40/91	44	89/816	10.9	Ziech et al. (77)
	PE	PCR^c^	Cow	6/21	28.6	7/383	1.8	Oliveira et al. (78)
	PB	PCR^c^	Cow	6/19	31.6	21/273	7.7	Filho et al. (79)
Argentina	La Pampa	DIF^a^	Bull	86/3766	2.3	437/29178	1.5	Molina et al. (80)
	Different regions	–	Bull	–	9,8-15,3	–	1–5	Campero et al. (36)
	La Pampa	DIF^a^	Bull	–/6000	3-10	–	–	Molina et al. (29)
Uruguay	National	DIF^a^ and I^b^	Bull	85/230	37	492/1754	28.05 and 12	Repiso et al. (49)
Colombia	Piemonte, Caribe, Andina	I^b^	Bull	–/113	19.4	–	15	Griffiths et al. (81)

*MS, Mato Grosso do Sul; MG, Minas Gerais; BA, Bahia; GO, Goias; MA, Maranhão; MT, Mato Grosso; PA, Pará; PR, Paraná; RS, Rio Grande do Sul; RO, Rondonia; SP, São Paulo; TO, Tocantins; RJ, Rio de Janeiro; PE, Pernambuco; PB, Paraíba*.

a Direct immunofluorescence;

b Isolation;

c *Polymerase Chain Reaction.–uninformed*.

The herd-level prevalence of BGC in different regions of the studied countries ranged between 2.3 and 100%. This dispersion is expected since the transmission of the disease depends on the sanitary and reproductive management practices used at each site (82). However, it is important to note that in many of these studies the differences could be associated with the diagnostic tests used. Most of the studies were based on DIF (Table 2) which does not distinguish the subspecies of *C. fetus* (31). Direct immunofluorescence should not be used as the unique diagnostic technique for confirmation of *Cfv* infection.

In Uruguay, a national study in beef cattle evaluated 1,754 samples of preputial smegma and determined a prevalence of 28% of positive bulls in 142 of 230 (37%) farms. The diagnosis was performed by DIF and bacterial culture directly from preputial smegma samples enriched in TEM for 72 h. In 47 of the farms the isolation of *C. fetus* was successful and 35 (75%) of these isolates were identified as *Cfv* (49). Culture and identification of *Cfv* reliably confirmed BGC in 15.2% of the farms, suggesting that the prevalence of 37% was likely overestimated. These variations may have occurred due to the differences between sensitivity and specificity inherent to the diagnostic tests used in different studies.

Several studies have also been carried out in different regions of Brazil, diagnosing BGC by DIF in up to 51.7% of the animals evaluated (20, 73–76). Rocha et al. (75) determined that the prevalence in bulls, by means of DIF, was 35.9%; however, it drop to 10.3% when using bacterial culture.

More recent works performed using PCR techniques that are more specific and sensitive than DIF and can differentiate between subspecies of *C. fetus* determined a lower prevalence of 7.7% (79) and 1.8% (78), in different regions of Brazil. Additionally, Filho et al. (79) confirmed the findings through whole genome sequencing of the strains.

Direct immunofluorescence can be used as a screening test for *C. fetus* (20, 49, 73–75, 80). However, DIF should not be used for the definitive diagnosis of *Cfv* infection. PCR-based methods and bacterial culture presented better results to estimate the prevalence of BGC (79, 81). However, a critical evaluation of clinical relevance is necessary when the diagnosis of BGC is established by phenotypic (63) and genotypic tests alone (83). On & Harrington (84) argue that the combination of identification methods (phenotype and PCR) mostly supports the taxonomic division of the species; however, the use of more than one method is necessary because the precise identification between subspecies is still problematic.

While reliable diagnostic tests for *Cfv* and *Cff* detection are not validated in South America and the real prevalence is not reported, recommending adequate control strategies is a difficult task (29). At this point, although DIF or PCR techniques can be used, they could be used as screening to detect *C. fetus* without disregarding isolation and identification. Isolation, in addition to still being essential for diagnosis, is also fundamental to identify the circulating strains and perform molecular studies, besides determining the possible role of *Cff* strains in the production of venereal disease.

## Conclusions

Bovine genital Campylobacteriosis is known to cause reproductive losses in various South American countries, however, these losses have not been quantified. The lack of widespread availability of simple, sensitive, and specific techniques for the laboratory diagnosis of *Cfv* infection, combined with limited laboratory infrastructure, insufficient resources for the development, and/or improvement of diagnostic tests, are key limiting factors for the diagnosis and control of BGC in the region.

Broader epidemiological studies to determine prevalence, risk factors and consequently estimate the economic losses of BGC in South America should be conducted. Because bulls are lifelong carriers of *Cfv* in the prepuce, they should be the sampling targets in BGC endemic herds and tested for *Cfv* with reliable screening and confirmatory diagnostic tests.

The combination of evidence increases the effectiveness of the results when correctly interpreted. Direct immunofluorescence and antigen capture ELISA determine the presence of *C. fetus*, being valuable for screening, but should be used in conjunction with bacterial isolation and phenotypic identification. PCR and/or DNA sequencing are valuable tools for confirming phenotypic tests. The isolation of *Cfv* is crucial to obtain autochthonous strains for future research and to develop and validate diagnostic techniques and reduce the cost of diagnosis. PCR-based methods appear to be promising for the diagnosis in field samples of preputial smegma, vaginal mucus, and tissues and/or fluids from aborted fetuses and/or placentas, as well as bacterial isolates. The use of these techniques for the unequivocal identification of *Cfv* and therefore the final diagnosis of BGC is recommended because they are more specific and sensitive and can differentiate between *C. fetus* subspecies. Veterinary diagnostic laboratories in South America should work toward accreditation and validation of diagnostic tests to provide reliable diagnostics to veterinary practitioners and farmers aiming at controlling or eradicating BGC from endemic herds and preventing the introduction of infected bulls in disease-free herds.

Although endemic, there are still no official guidelines regarding BGC control in South American herds. The development of regional health policies with monitoring and surveillance would help to establish control and eradication programs.

## Author Contributions

CS: first author who was responsible for the search of the works, organization and writing of this manuscript; MF: contribution in the revision of the manuscript, especially in what concerns molecular biology and language; FG: contribution in the revision of the manuscript and language; MM-R: contribution in the revision of the manuscript and language; FR-C: Last author and coordinator, his contribution was essential for the completion of this work, applied his corrections and critical reviews in the theoretical part and in the language. All authors have seen and approved the content of the manuscript and have contributed significantly to the work.

### Conflict of Interest Statement

The authors declare that the research was conducted in the absence of any commercial or financial relationships that could be construed as a potential conflict of interest.
